# Vibration Damping Analysis of Lightweight Structures in Machine Tools

**DOI:** 10.3390/ma10030297

**Published:** 2017-03-15

**Authors:** Francesco Aggogeri, Alberto Borboni, Angelo Merlo, Nicola Pellegrini, Raffaele Ricatto

**Affiliations:** 1 Department of Mechanical and Industrial Engineering, University of Brescia, via Branze, 38, 25123 Brescia, Italy; alberto.borboni@unibs.it (A.B.); nicola.pellegrini@unibs.it (N.P.); 2 CE.S.I Centro Studi Industriali, via Tintoretto, 10, 20093 Cologno Monzese, Italy; merlo@cesi.net; 3 FIDIA Spa, c.so Lombardia, 11, 10099 Torino, Italy; r.ricatto@fidia.it

**Keywords:** hybrid materials, machine tool structures, modal analysis, machine tool kinematics, aluminium metal foams, aluminium corrugated sandwiches, CFRP materials, FE simulations, damping

## Abstract

The dynamic behaviour of a machine tool (MT) directly influences the machining performance. The adoption of lightweight structures may reduce the effects of undesired vibrations and increase the workpiece quality. This paper aims to present and compare a set of hybrid materials that may be excellent candidates to fabricate the MT moving parts. The selected materials have high dynamic characteristics and capacity to dampen mechanical vibrations. In this way, starting from the kinematic model of a milling machine, this study evaluates a number of prototypes made of Al foam sandwiches (AFS), Al corrugated sandwiches (ACS) and composite materials reinforced by carbon fibres (CFRP). These prototypes represented the *Z*-axis ram of a commercial milling machine. The static and dynamical properties have been analysed by using both finite element (FE) simulations and experimental tests. The obtained results show that the proposed structures may be a valid alternative to the conventional materials of MT moving parts, increasing machining performance. In particular, the AFS prototype highlighted a damping ratio that is 20 times greater than a conventional ram (e.g., steel). Its application is particularly suitable to minimize unwanted oscillations during high-speed finishing operations. The results also show that the CFRP structure guarantees high stiffness with a weight reduced by 48.5%, suggesting effective applications in roughing operations, saving MT energy consumption. The ACS structure has a good trade-off between stiffness and damping and may represent a further alternative, if correctly evaluated.

## 1. Introduction

The dynamic behaviour of a machine tool (MT) plays a crucial role in satisfying the main machining requirements, like high-speed operations, precision in axis positioning, and the capability to quickly remove a high quantity of workpiece material [1]. These performances are directly related to the materials used in MT construction. For this reason, materials of MT foundations and moving parts need to be selected with high dynamic characteristics and capacity to dampen mechanical vibrations.

The MT structures guarantee withstanding the forces generated by the process and the machine motions [2]. Forces acting on the MT structure during motion represent a serious limitation for the precision and productivity and, therefore, they are evaluated during machine design and material selection [3,4]. In particular, structural deformations may lead to unwanted displacements of the tool tip point (TTP), causing undesired deviations and degradations of surface quality during finishing. In order to avoid these undesirable effects, the adopted materials must satisfy a set of requirements, such as high static stiffness for bending and torsion, a high value of elastic modulus, yield strength, good dynamic characteristics, and dimensional stability [5].

These reasons have motivated researchers to study and design lightweight mobile parts of MTs, increasing stiffness and capacity in vibration damping and guaranteeing the required precision. Specifically, the challenge is to identify the proper, and cost effective, structural materials that are able to meet the requirements in terms of productivity, accuracy, and eco-efficiency [6].

This paper aims to evaluate the static and dynamic performances of a set of MT structures made of hybrid materials. In particular, this work focuses on the capacity of a mobile part to guarantee high-speed operations to reduce undesired effects. In fact, high cutting and transfer speeds represent essential requisites to satisfy productivity and sustain competitiveness. Productivity is often limited by low transmission speeds of moving parts, generally made of cast iron. Modern machines need to manage accelerations of 14 m/s^2^ and speeds from 3 m/s [7]. The utilization of large quantities of steel may generate undesired vibrations in high transfer speed conditions, seriously compromising the quality of the workpiece [8,9]. In this way, hybrid materials that are able to dampen mechanical vibrations may be preferred in manufacturing MT structural parts. 

Vibrations are undesired effects that may influence the quality of a workpiece. Vibrations are usually considered in the design phase of a MT, analysing the structure conception in terms of mass reduction, stiffness increase, and material selection. In MTs, three distinctive types of vibrations are noted and classified: forced, self-generative, and free vibrations [10,11]. The first type of vibration is generated by periodic forces arising within the machine. These vibrations may originate from multiple events, such as intermittent cutting, unbalanced rotating components, spindle or gear wear-out issues, or unstable effects. The state of the art shows a number of solutions that allows limiting this effect (e.g., active vibration control, AVC systems; mechatronic models based on compensation, as shown in Table 1). The second class of vibrations—self-generative—may occur as a result of some processes in the machine. In particular, one of the main undesirable dynamic process phenomena is machine tool chatter. This is generated by the interaction between the machine structure and the cutting process. These vibrations have frequencies in the range from 200 to 1000 Hz. This effect may be mitigated by optimizing spindle speed or depth of cut. An alternative solution is the use of active structural control systems to alter the dynamics by installing actuators or sensors in a closed-loop. The last group of vibration is related to free vibrations. These vibrations originate from material imperfections, inertial forces, or shocks from MT basements. They occur during strong accelerations, when inertial forces generate oscillations, causing geometrical inaccuracies in the workpiece. In this case, the selection of stiff and light-weight materials may improve the machining performance, limiting vibration issues.

From a practical point of view, all of these vibrations have equal importance on workpiece quality, machining performance, and accuracy. The state of the art presents a set of strategies that may be adopted to mitigate the vibration effects. These techniques aim to compensate vibrations using passive or active control strategies. Table 1 summarizes a set of studies that suggest different approaches in MT vibration control and mitigation. 

The current state of the art shows examples and applications of MT structures made of hybrid materials [12,13,14,15,16,17], nevertheless these studies do not offer a complete comparison between materials, based on the static and dynamic performances of the mobile parts. The modern machines need to satisfy high and strict requirements in terms of precision, speed, and flexibility. These performances are directly related to the materials used in MT construction; the push of new market needs versus new solutions and applications. The proposed structures may represent an effective answer to satisfy these requirements.

The new contribution of this paper is to provide a detailed analysis of a set of structures made of hybrid materials. In particular, Al foam sandwiches (AFS), Al corrugated sandwiches (ACS), and composite materials reinforced by carbon fibres (CFRP) are considered. These materials have been selected as valid alternatives to the conventional approaches since they have good structural and damping properties. The scope is to underline advantages and limits of the proposed structures, suggesting the industrial applications. For this reason, a performance comparison of a set of prototypes is presented. These prototypes represent the *Z*-axis ram of a commercial milling machine. The choice to use real, oversized MT parts allowed the evaluation of the effect of all potential variables on the structural behaviour. These variables are not only the material properties, but also the technical decisions, the design constraints, and the technological limits that might be accurately analysed using the real prototypes. 

Starting from the kinematic analysis of a milling machine that highlighted the main critical factors to damp vibrations, the static and dynamic properties have been analysed by using both finite element (FE) simulations and experimental tests. In particular, FE simulations allowed to improve the structure design, increasing structural indices and avoiding technical issues in prototype fabrication. The sample performances have been evaluated and compared through static and dynamic tests. The proposed solutions have been studied considering the conventional materials (e.g., steel, cast iron), usually adopted in MT structure fabrication. 

**Figure 1:**
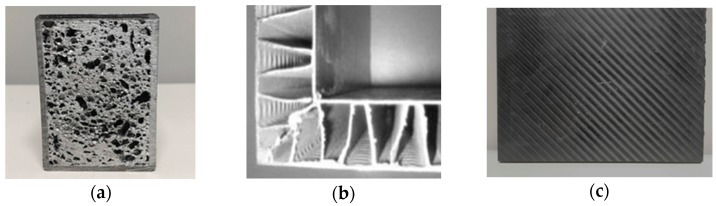
Lightweight MT structure materials: Al foam sandwiches (AFS) (**a**); Al corrugated-core sandwiches (**b**); and composite materials reinforced by carbon fibres (CFRP) (**c**).

Figure 1 illustrates an overview of the selected hybrid materials. Metal foams represent an innovative category of materials. They have low density with good shear and fracture strength. Metal foams guarantee both lightweight and stiff structures with low costs [33,34]. These performances are reached through the novel mechanical, thermal, and physical properties.

Corrugated-core sandwiches are another valid alternative to guarantee bending stiffness and strength when minimal mass is required. These Al sandwich panels are structurally lightweight, offering 30%–40% of weight savings compared with conventional structures. The lightness of the panels is achieved through the layer build-up, which permits incremental wall thicknesses of the structure. They have a broad range of applications; in particular they are suitable to improve the stiffness and reliability of constructions, absorbing energy during blasts and impacts [35,36,37,38,39]. The composite materials reinforced by carbon fibres (CFRP)) are particularly interesting for applications in machine tool structures due to their ratio of mechanical strength to density [40,41]. CFRP materials consist of a binding matrix system and a reinforcement of fibres or particles.

The selected CFRP materials have fibres with diameter of 4–10 μm. The matrix is made of either polymer resin and epoxy, and polyimide. The characteristics of fibres (strength, orientation, length) and matrix (volumetric content, layer) define the mechanical properties of the CFRP materials. In addition, the composites reinforced by unidirectional fibres have the highest mechanical performance in fibre direction. 

## 2. Materials and Methods

The strategy to select the most suitable materials is based on the analysis of a set of parameters. These parameters consist of structural and damping indicators. In particular, the method suggested by Ashby is applied, focusing on material weight reduction and stiffness increase [42,43,44]. The main structural index is described by Equation (1):

(1) $$C=E^{1/3}/\rho ,$$

where *E* is the Young’s modulus and ρ is the material density. This indicator links the material mass and stiffness. In this way, the weight is reduced and the stiffness is increased by selecting materials with high values of this structural index. A further indicator is the loss factor (hysteretic damping) that represents the attitude of material to dampen vibrations [45]. Table 2 lists the main values of these indicators, comparing the conventional materials with a set of potential hybrid materials, used for MT moving part fabrication. 

This preliminary investigation shows that the CFRP materials have the highest structural index. In the same way, Al foams and Al corrugated sandwiches highlight good structural and damping properties and they may be a further suitable solution to satisfy the structure requirements. 

Table 2 also underlines the excellent dynamic properties of Mg alloys; nevertheless, these materials are expensive due to the complexity of their fabrication processes. For this reason they are not included in this study. Starting from these considerations, the selected solutions represent a great challenge, opening interesting perspectives for MT applications.

### 2.1. Analysis of Vibration Damping in Machine Tools

Vibration damping analysis is one of the most critical activities in MT design. For this reason, it is useful to study the static and dynamic behaviour of the machine structures using a parametric model. This model is evaluated in a simplified form to provide a better understanding of experimental tests [1]. In particular, it is suitable to represent the kinematics of a number of machines, such as gantry or travelling column machines (e.g., milling or boring machines). As defined by Apprich et al. [46], this model depends on movement, position, and type of machining. For this reason, the main parameters need to be adjusted in real-time in order to compensate for vibrational effects. Nevertheless, the kinematic representation of a general machine permits understanding of the main critical variables in designing effective lightweight structures without sacrificing the stiffness. 

**Figure 2:**
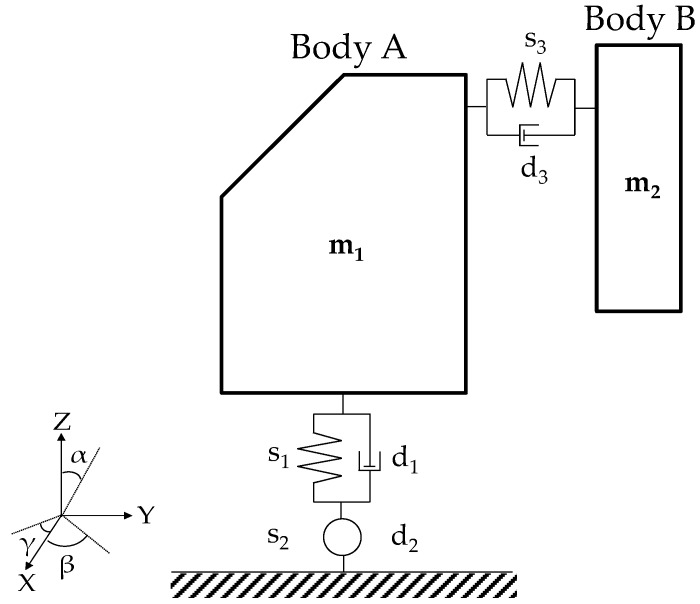
General machine kinematic model.

The presented kinematic model is developed on the machine configuration described by Figure 2. It is assumed that the machine may be represented as two rigid bodies: body A, that is, the MT moveable frame, and body B, which represents the *Z*-axis ram.

Body A, with the mass equal to *m*_1_, has three rotational degrees of freedom (DOFs) constrained to the basement. These DOFs allow evaluating its natural frequencies. In this way, a simple finite segment method (FSM) may model the stiffness using one rigid mass and three springs for each degree of freedom. This approach is based on modelling a flexible body as a finite number of rigid elements that are linked by dampers and springs. This requires the use of rigid multibody formulations. The spring s_1_ defines the bending in the *Z*-axis, s_2_ describes the torsion around the *Y*-axis, while s_3_ shows the inflection in the *X*-axis. The dampers *d*_i_ (i = 1, 2, 3) regulate the resonance, without influencing the natural frequencies of the body. It is assumed that the translational movement of body A is not permitted. 

Body B, with mass equal to m_2_, is the ram of the machine tool. It has a translation DOF in the *Z*-axis that represents the rigid movement of the slide. It also has a DOF, defined by s_3_ and d_3_ in the *Y*-axis.

Assuming that a set of external forces (F_x_, F_y_, F_z_) is applied to the machine ram (body B), the dynamic behaviour of the system may be evaluated by applying the Newton-Euler equations for a general coordinate *z* = [*α*, *β*, *γ*, L_Y_, L_Z_], where L_Y_ and L_Z_ are the distances between the body’s barycenters (in the *Y*-*Z* plane).

By using the D’Alembert’s principle for dynamic equilibrium conditions in Lagrange’s form, the multibody system is described by Equations (2) and (3), as follows [46,47]:

(2) $$M(u,t)\cdot \ddot{z}+k(u,\dot{u},t)=q(u,\dot{u},t),$$

(3) $$\sum_{i=1}^{p}\left[J_{T_{i}}^{T}\cdot m_{i}\cdot J_{T_{i}}+J_{R_{i}}^{T}\cdot I_{i}\cdot J_{R_{i}}\right]\cdot \ddot{z}+\sum_{i=1}^{p}\left[J_{T_{i}}^{T}\cdot m_{i}\cdot {\bar{a}}_{i}+J_{R_{i}}^{T}\cdot I_{i}\cdot {\bar{a}}_{R_{i}}+J_{R_{i}}^{T}\cdot {\tilde{\omega }}_{i}\cdot I_{i}\cdot \omega_{i}\right]=\sum_{i=1}^{p}\left[J_{T_{i}}^{T}\cdot f_{i}+J_{R_{i}}^{T}\cdot h_{i}\right],$$

where

- $J_{R_{i}},J_{T_{i}}$: Jacobian matrices of rotation and translation of the *i*-th body,
- $I_{i}$: Inertia matrix of the *i*-th body,
- ${\bar{a}}_{i}$: Acceleration of the *i*-th body,
- $\omega_{i}$: Angular rate of the *i*-th body,
- ${\tilde{\omega }}_{i}$: Rotation matrix of the *i*-th body,
- $f_{i},h_{i}$: External forces and moments of the *i*-th body,
- *m*: Mass matrix

In this way, a general coordinate *z* of the multibody system is expressed by a number of *f* differential equations, where the movement of the machine ram is represented by the inertia matrix and moments *l_i_*, acting on the body. In particular, the model parameters (e.g., Jacobian matrixes, speeds, accelerations) depend on the position and they need to be calculated from theoretical considerations. However, it is also interesting to highlight that the active forces are not pose-dependent [41,48]. This parameter model may be useful both for the numerical studies (e.g., finite element analysis—FEA) and for the experimental campaigns (e.g., static and dynamic tests). It underlines those variables that describe the machine dynamic behaviour and, as a consequence, how to compensate for the undesired effects of vibrations. For example, assuming a prescribed mass to guarantee the lightweight characteristics of a structure, it is noted that the stiffness may be improved through the increase of the structure inertia, working on ram thicknesses and dimensions. This study may be developed using FE simulations that produce an accurate representation of distributed stiffness and inertia properties.

### 2.2. The Lighweight Structure Prototypes 

**Figure 3:**
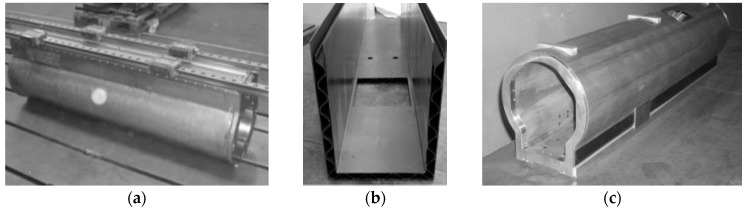
Lightweight MT structure prototypes: Al metal foams (AFS) (**a**); Al corrugated-core sandwiches (ACS) (**b**); and carbon fibre reinforced polymer (CFRP) (**c**).

In order to compare the lightweight structures, a set of prototypes was fabricated. The prototypes represented the *Z*-axis ram (300 mm × 1500 mm) of a commercial milling machine and they were made of aluminium foam sandwiches, in closed-cell configuration, corrugated-core sandwiches, and CFRP material, respectively. Figure 3 shows an overview of the three prepared samples. The prototype dimensions were designed satisfying the MT requirements, guaranteeing that natural MT vibration frequencies passed at least 50 Hz. The vertical axis prototypes were equipped by commercial guideways, reproducing the same characteristics of the final version of a machine tool element. A number of structural versions were studied using FE simulations in order to satisfy the required structural properties. Figure 3 illustrates the final prototype design validated by the finite element analysis (FEA), avoiding any potential technical issues or defects and, as a consequence, an increase of the experimental campaign costs. 

## 3. The Stiffness Analysis of the Lightweight Structures 

### 3.1. Numerical Analysis of the Structure Stiffness 

To investigate and compare the performance of the selected materials, the first phase of this study evaluated the structural behaviour when a static load was applied, using a set of simulations. A model was built by a finite element analysis (FEA) that was able to represent the mechanical properties of the structures. This analysis was executed on a ram of a commercial milling machine. In particular, the simulations wanted to compare the conventional material (e.g., steel) with the selected materials, modelling a set of external loads that may usually occur during the working operations. The main scope was to evaluate the static performance of the lightweight structures and to provide a preliminary assessment of the prototypes design. In fact, the simulations considered the material mechanical properties, dimensions and weight. The kinematic behaviour was modelled by commercial FE software.

**Figure 4:**
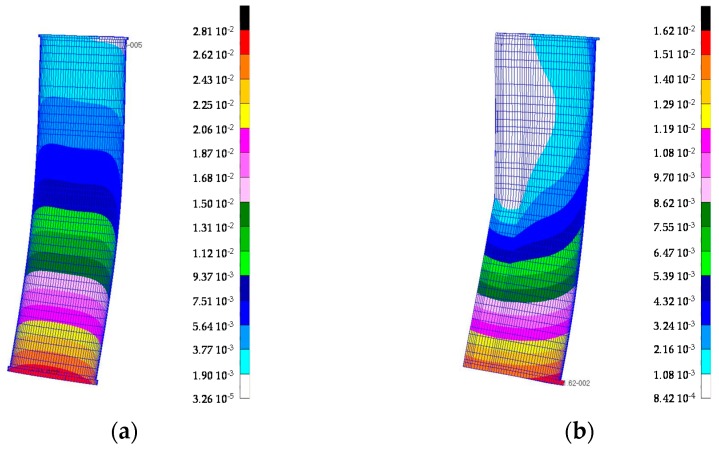
FE model of AFS structure: the deformed shape (μm) in the *X* orientation (**a**) and in *Y* orientation (**b**), applying a static load of 60 N at tool tip point.

Figure 4 shows an example of FE analysis performed on the final design of aluminium foam sandwich (AFS) structures. A set of loads was applied at the TTP of the ram and the stiffness was calculated in the *X*, *Y*, and *Z* directions. The effect of gluing was not considered for both the AFS sample and the Al corrugated sandwich sample. Table 3 lists the mass variation between the proposed samples and a conventional ram. The conventional ram was made of steel and its weight was equal to 97 kg.

Table 4 illustrates the results obtained from the FEA simulations. It is noted that all materials show a better performance than the conventional ram (steel) both in the *Y* and *Z* directions. In particular, the AFS ram has the highest stiffness in the *Z* direction, its performance is greater than the convention material (steel) by 152%. Nevertheless, it is the heaviest structure, as shown in Table 3. This fact, considered in the simulation, is due to the oversizing of sample ribs and flanges required by the prototype installation. 

The CFRP structure has the best stiffness in the *Y* direction, with a significant saving of weight (48.5%), whereas the Al corrugated sandwich ram exhibits some issues in terms of stiffness in the *X* direction. This weakness is due to the structural configuration; however, a suitable stiffness is guaranteed both in the *Z* and *Y* axis.

In any case, the FEA results needed to be validated and confirmed by the experimental tests. In fact, the characterization of samples has been a complex process since these materials have isotropic and stochastic features. Their characteristics may change based on manufacturing processes or chemical composition. For this type of hybrid materials, a simulation error is expected lower than 10%–15% of the real value. This error may be considered acceptable since the cutting forces are usually lower than 100 N. This means a maximum simulation error of the tool tip displacement equal to ±2 μm.

### 3.2. Experimental Test Campaign: The Stiffness Analysis of the Lightweight Structures

An experimental campaign has been performed to validate the results of the simulations. As shown, the structure stiffness plays a crucial role to satisfy the MT requirements.

The structure stiffness was evaluated by applying a set of external forces in the *X* and *Y* directions, which were the most critical directions to guarantee the machining quality. 

**Figure 5:**
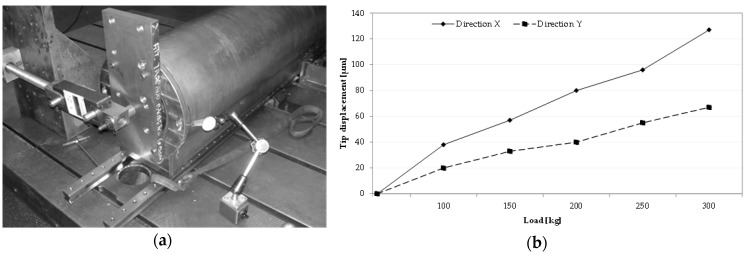
Test bench setup overview (**a**) and the experimental results of the statistic tests performed on a ram (**b**).

The experimental procedure consisted of controlling the static force by acting on a screw, avoiding peak or step stress. The corresponding deflections were measured at the tool tip point by a number of dial gauge sensors. Finally, the stiffness was quantified, calculating the ratio between the force and the corresponding displacement at the application point. Figure 5a illustrates an overview of the test bench used to execute the static tests. The applied forces varied over a range from 10 kg to 300 kg. Figure 5b highlights an example of the results obtained from the AFS ram.

In order to confirm the numerical analysis, a comparison between simulations and experimental tests was evaluated. The experimental stiffness has been compared with the simulated results in the *X* and *Y* directions. Table 5 shows a substantial matching between the FE models and the experiment tests, with a maximum error of 15%. As expected, the AFS and CFRP structures were the most critical rams to be characterized, since they had a complex internal configuration that depends of the prototype fabrication process. This point allowed reviewing and improvement of the FE model in order to develop further simulations (e.g., modal analysis and dynamical structure behaviour), as shown in Section 4.

Table 5 summarizes the comparison between the lightweight structures and the conventional ram (steel). CFRP ram shows the best performance in both directions (*X*-*Y*), while the Al corrugated sandwich structure has some issues in the *X* direction due to the structural configuration. 

This preliminary investigation underlines that the selected lightweight materials are good candidates to be used in MT moving part fabrication. They have suitable mechanical properties to guarantee the machining performance in terms of stiffness. In particular, a significant saving of weight is noted both for CFRP and Al corrugated sandwich structures. The AFS ram has the best trade-off of stiffness in the *X* and *Y* directions; nevertheless, some limitations in terms of weight are observed due to the oversizing of sample ribs and flanges, required for the prototype installation. A design review may solve this problem.

## 4. The Modal Analysis of the Lightweight Structures 

A modal analysis was performed to study the dynamic characteristics of the structures under vibrational excitation [49,50,51,52]. To characterize the damping capacity of a hybrid material, it is noted that the damping characteristics of viscoelastic materials are dependent on frequency and they are defined by the variable complex modulus T*(*ω*) [53,54,55], as shown in Equation (4):

(4) $$T*(\omega )=T^{\prime }(\omega )+i\cdot T^{\prime \prime }(\omega )=T(\omega )\cdot (1+i\cdot \eta (\omega )),$$

where *T*(*ω*) = *T’*(*ω*) is the storage modulus, *T’’*(*ω*) is the loss modulus and *η*(*ω*) is the loss factor that is defined by the Equation (5):

(5) $$\eta (\omega )=\frac{T^{\prime \prime }(\omega )}{T^{\prime }(\omega )},$$

The storage modulus *T*(*ω*) and the loss factor *η*(*ω*) depend on the excitation frequency and they are the properties which distinct the viscoelastic damping to the other damping mechanisms [56,57,58]. The characterization of the complex modulus plays a critical role in MT material selection, since it allows knowing the bandwidth of the viscoelastic materials. The half-power bandwidth procedure is an effective method for damping identification that defines the modal loss factor *η*(*ω*) as the relationship between the frequency range ∆*ω* over which the response diminishes 3.01 dB with respect to the maximum level given at resonance frequency. In the case of multiple degrees of freedom, the calculation is more complex and the use of further techniques is required [51].

**Figure 6:**
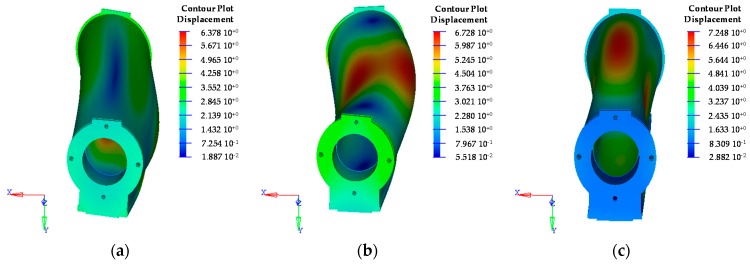
The FE modes 1–3 at 650 Hz (**a**); 677 Hz (**b**); and 709 Hz (**c**) performed on the AFS ram.

A dynamic simulation of the structures has been performed and compared with the experimental modal analysis. To understand the adopted approach, the complete study is only described for the AFS structure. The simulation results of the free-free modal analysis are listed in Table 6 and Figure 6. 

To confirm the simulations, an experimental modal analysis was executed using the “hammer test”. This experimental campaign was executed on the structures (conventional and hybrid material rams) in order to compare the dynamic properties. A test bench was equipped with an accelerometer to measure the response acceleration at a fixed point and direction, a four-channel FFT (fast Fourier transformation) analyser to compute frequency response functions (FRFs), a hammer, with a load cell to measure the input force, a post-processing modal software for identifying modal parameters and displaying the mode shapes, and a system able to suspend the prototype in free-free configuration. The prototypes were excited by a hammer in two different directions and positions in order to have a complete modal response.

**Figure 7:**
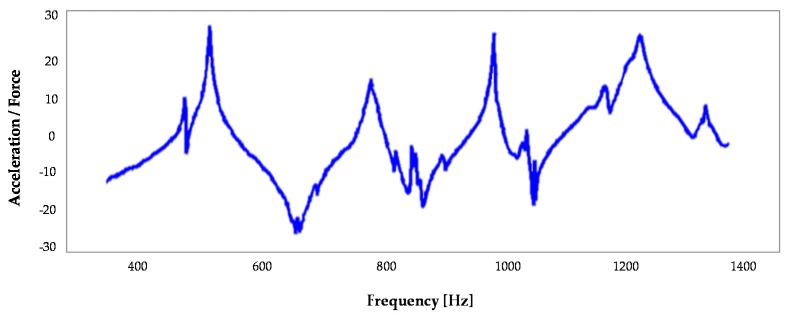
The frequency response function (FRF) of the excited point (centre of ram) in the *Y* direction.

Figure 7 illustrates the frequency response function (FRF) of the AFS structure. FRF is a transfer function, expressed in the frequency domain. It defines the structural response to the applied force as a function of frequency, describing the relationship between the displacement and the point of the applied load (centre of the ram). A preliminary investigation shows a mismatching of the first modes between the numerical analysis and the experimental tests. In fact, the first two modes of the tests occur at 441 Hz and 483 Hz, while the FE simulation highlighted these modes at 650 Hz and 677 Hz. This fact is due to the constraints’ interaction with the support structure of the test bench and they could not be considered in the simulation. In any case, these modes are not important for the scope of this study; in fact they are breathing modes and do not influence the tool tip displacement. 

In order to evaluate the AFS structure damping, there is a good agreement between the simulations and experimental results for bending and torsional modes, which are critical in MT vibration damping. In particular, it is noted the bending mode in the *X*-*Z* plane at 667 Hz, the bending mode in the *Y*-*Z* plane at 758 Hz and the torsion mode at 816 Hz, as also shown in the simulation (Figure 6). They appear with a limited peak, highlighting the high capacity of the material to dampen the vibrations. The loss factor *η* has been calculated for each mode, underlining an important growth of the damping performance, coinciding with the inflectional and torsional mode of the *Z* axis. The AFS loss factor of the first modal frequency (bending) was equal to 1.7%. In the same way, the study has been replicated for all prototypes. Table 7 lists the loss factor of the lightweight structures and the conventional ram. It is noted that the hybrid materials have significant dynamical properties to reduce the vibration effect. The AFS material has a damping capacity that is 20–30 times greater than a conventional structure. The CFRP structure has good damping properties, achieving the first modal frequency close to 1200 Hz. 

## 5. Discussion 

**Figure 8:**
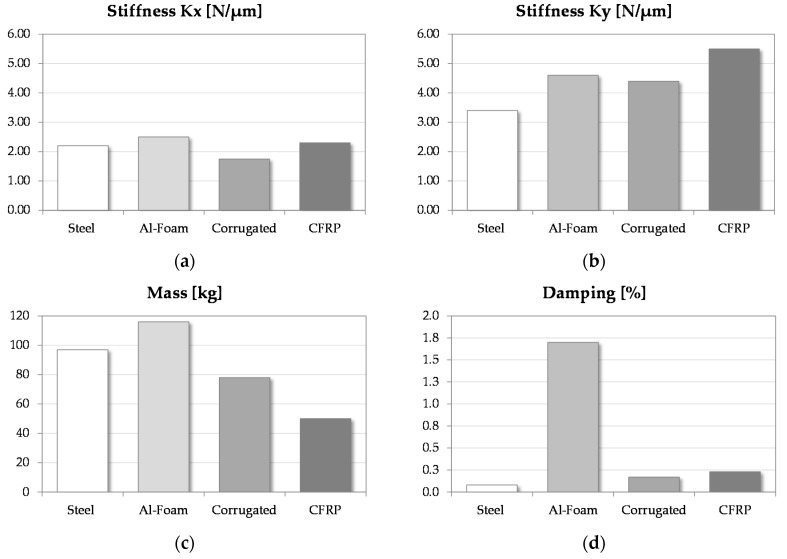
The result comparison: *X*-axis stiffness (**a**); *Y*-axis stiffness (**b**); mass (**c**); and damping (**d**).

These materials are excellent candidates to substitute the conventional structures (e.g., cast iron or steel). In fact, they guarantee high mechanical properties and high dynamic characteristics to dampen vibrations. Figure 8 summarizes the comparison between the lightweight structures and the conventional ram (steel).

The experimental tests show that the AFS structure has a stiffness greater than the conventional ram stiffness of 152.7% in the *Z* axis, 11.4% in the *X* axis, and 14.7% in the *Y* axis. This point guarantees withstanding the forces generated by hard machining and machine motions. This structure also has a high capacity to dampen the vibrations. In fact, the loss factor is 20 times higher than the factor of the other evaluated materials. The main limit is represented by the weight. In this study, the AFS prototype was the heaviest structure due to the particular configuration to be installed in the milling machine. Nevertheless, a design review may solve this problem, obtaining weight savings. These results suggest the use of AFS structures when it is required to minimize unwanted oscillations during high-speed finishing operations. 

In the same way, the CFRP ram highlights good mechanical characteristics in terms of stiffness and damping. The main advantage is the saving of weight, which is 48.5% less than steel. This solution may be preferred to conventional structures to quickly remove hard workpiece materials (e.g., titanium alloys or hardened steels) in roughing operations, saving energy consumption.

Finally, the Al corrugated-core sandwiches are a further alternative to fabricate the MT moving parts. This structure underlines a good trade-off between the mechanical properties and the cost of fabrication. Nevertheless, some issues of stiffness have been noted in the *X* direction due to the sandwich configuration. They are very cost-effective materials and may represent a further alternative, if correctly evaluated.

## 6. Conclusions 

This paper deals with the dynamic behaviour of machine tool structures, focusing on the evaluation of the main properties of a set of lightweight materials. These materials may represent a valid alternative to conventional structures, which are often unsuitable to satisfy the machining requirements. In fact, modern machines need to satisfy high and strict requirements in terms of operation speed, precision, quality, and capability to quickly remove high quantities of workpiece material. These performances are directly related to the materials used in MT construction. 

This paper offers a complete evaluation of a set of hybrid materials to fabricate mobile parts, improving machining performances. The application of these materials may open new perspectives to satisfy the market requirements. Literature shows a few, and limited, examples of hybrid materials in MT structure fabrication; nevertheless, the MT market pushes towards new solutions and applications to guarantee machining precision, speed, and flexibility. The proposed structures may represent an excellent alternative to conventional materials and an effective answer to satisfy these market requirements. The main application limits, such as fabrication complexity and costs, may be solved through an effective structure design and simulation. 

This paper analyses a number of samples made of Al foam sandwiches, Al corrugated sandwiches, and CFRP materials, evaluating the structural performances in terms of static and dynamical properties. The fabricated prototypes represent the *Z*-axis ram (300 × 1500 mm) of a commercial milling machine. The first part of the study illustrates the hybrid materials properties, in terms of structural indexes and damping capacity. In order to study all potential variables that may impact on the static and dynamic behaviour of the structures, the prototypes have been accurately analysed through FE simulations and static tests. The obtained results confirmed the saving of weight of the structures made of hybrid materials and the high stiffness, compared to conventional materials (e.g., steel, cast iron). The second part of the paper focuses on the modal analysis. A set of simulations was executed to understand the main vibration modes of the lightweight structures. Then, an experimental campaign was executed through the “hammer test” in order to study the dynamic behaviour. The AFS ram shows a damping that is 20 times greater than the conventional ram. These results suggest to use these structures in high-speed finishing operations, guaranteeing the workpiece quality and high productivity. 

The other hybrid materials highlight good capacities to dampen vibrations. In particular, the high stiffness of the CFRP structures allow effective applications in roughing operations to remove hard workpiece materials, saving MT energy consumption. 

Finally, some limits are noted in terms of fabrication complexity and costs. Nevertheless, these problems may be limited and solved through an effective design of the structures, using simulations and experimental tests to optimize the weight and, as a consequence, the costs. 

## Figures and Tables

**Table 1:** Active and passive methods to control and mitigate MT vibrations.

Vibration Damping	Characteristics	References
Active methods	Installation of additional devices, such as actuators Use of advanced and complex control algorithms Knowledge of vibrations’ eigen-frequencies Model-based strategy	[1,18,19,20,21,22,23,24]
Passive methods	Based on viscoelastic materials, viscous fluids, magnetic or passive piezoelectric, lightweight materials Vibration energy dissipation or redirection Cost effective Dampers are usually small size and easy to install	[16,25,26,27,28,29,30,31,32]

**Table 2:** Material comparison: structural index versus loss factor.

Material	*E*^1/3^/*ρ* (GPa^1/3^/(Mg/m^3^))	*η*
Cast iron	0.63	1.2 × 10^−3^–1.7 × 10^−3^
Steel	0.77	6.0 × 10^−4^–1.0 × 10^−3^
Al alloys	1.50	2.0 × 10^−4^–4.0 × 10^−4^
Mg alloys	1.90	1.0 × 10^−3^–1.0 × 10^−2^
Al corrugated sandwich	2.52	1.0 × 10^−3^–1.0 × 10^−2^
Al foams	2.67	4.0 × 10^−3^–1.0 × 10^−2^
CFRP (unidirectional)	4.00	1.5 × 10^−3^–3.0 × 10^−3^

**Table 3:** The structure mass comparison.

Configuration	Mass (kg)	Conventional Mass (kg)	Mass Variation (%)
AFS ram	116.0	97.0	+19.6%
Al corrug. ram	78.0	97.0	−19.5%
CFRP ram	50.0	97.0	−48.5%

**Table 4:** FE results: the structure stiffness comparison in the *X*, *Y*, and *Z* directions.

Configuration	Kx (kg/µm)	Ky (kg/µm)	Kz (kg/µm)
Conventional (steel) RAM	2.20	3.43	22.05
AFS RAM	2.48	3.95	55.62
Al corrug. RAM	1.90	4.41	42.81
CFRP RAM	2.65	5.10	39.85

**Table 5:** Static test results: a comparison of the structure stiffnesses.

Configuration	Direction	K (N/µm)	Simulation Error	Stiffness Comparison with a Conventional RAM
AFS ram	*X*	2.50	0.80%	+13.64%
	*Y*	4.64	14.87%	+35.29%
Al corrug. ram	*X*	1.75	8.57%	−20.45%
	*Y*	4.44	0.63%	+29.43%
CFRP ram	*X*	2.30	14.97%	+4.78%
	*Y*	5.55	8.09%	+61.38%

**Table 6:** Numerical modes and frequencies of the AFS ram.

Mode	FE Model Frequency (Hz)	Description
**1**	650	Breathing
**2**	677	Breathing and Bending *Z*-*X*
**3**	709	Breathing
**4**	782	Bending mode *Z*-*Y*
**5**	816	Torsion *Z* axis

**Table 7:** The loss factor of the selected structures.

Configuration	1st Frequency (Hz)	Loss Factor (%)
Conventional (steel) RAM	670	0.08
AFS RAM	667	1.70
Al corrug. RAM	745	0.17
CFRP RAM	1286	0.23
